# Probing molecular interactions of cellulose fibers with isomeric deep eutectic solvents using NMR spectroscopy

**DOI:** 10.1007/s10570-025-06913-0

**Published:** 2026-01-22

**Authors:** Mohan Rangaswamy, Yashu Kharbanda, Otto Mankinen, Juho Antti Sirviö, Sarah E. Mailhiot, Mehmet Zafer Köylü, Mateusz Urbańczyk, Henrikki Liimatainen, Ville-Veikko Telkki

**Affiliations:** 1 NMR Research Unit, University of Oulu, P.O. Box 3000, N90014 Oulu, Finland; 2 Fibre and Particle Engineering Research Unit, University of Oulu, P.O. Box 4300, 90014 Oulu, Finland; 3 INRAE, UR OPAALE, 17 Avenue de Cucillé, CS 64427, 35044 Rennes Cedex, France; 4 Department of Physics, Faculty of Sciences, Dicle University, Diyarbakir, Turkey; 5 Institute of Physical Chemistry, Polish Academy of Sciences, Warsaw, Poland

**Keywords:** Deep eutectic solvent, Pyrazole, Imidazole, Nuclear magnetic resonance, Swelling

## Abstract

Deep eutectic solvents (DESs) are promising media for cellulose fiber (CF) modifications, such as swelling, dissolution, functionalization, and disintegration, owing to their low toxicity, biodegradability, and versatility. These modifications are governed by CF–DES interactions, regulated by the chemical structures of DES constituents. Here, nuclear magnetic resonance (NMR) spectroscopy was used to investigate the molecular interactions and dynamics between CFs and two isomeric non-derivatizing DESs comprising triethylmethylammonium chloride (TEMACl) with imidazole (Imi), TEMACl–Imi, or TEMACl with pyrazole (Pyra), TEMACl–Pyra. The NMR approach encompassed variable-temperature ^1^H diffusion, *T*_1_-and-*T*_2_-relaxation, and ^13^C NMR experiments. Significant CF swelling occurred in TEMACl–Imi, highlighted by reduced Imi and almost unchanged TEMACl relaxation times. This indicated that Imi primarily accounted for the interaction with cellulose. Correspondingly, the diffusion coefficients (*D*) of both DES components reduced, probably because of the increased viscosity due to CF swelling, as well as restricted diffusion inside the swollen CFs. Further, the ^13^C NMR spectra displayed characteristic cellulose-backbone signals, indicating a swelling-induced increase in CF mobility. Conversely, TEMACl–Pyra exhibited significantly suppressed changes in ^1^H-relaxation times and *D*, and no cellulose signal appeared in its ^13^C spectra. This indicated suppressed CF swelling and CF–DES interaction. The more significant CF swelling in TEMACl–Imi might stem from the formation of stronger hydrogen bonds by Imi with the hydroxyl groups in cellulose compared with those formed by Pyra. Overall, these findings highlight how the molecular configuration of DES facilitates cellulose interactions and the profound impact of these interactions on CF modification.

**Supplementary Information:**

The online version contains supplementary material available at 10.1007/s10570-025-06913-0.

## Introduction

Cellulose-pulp fibers function as abundant, renewable bioresources for developing diverse sustainable materials and applications, ranging from traditional paper and board products to modern advanced designs, such as biodegradable plastics, smart textiles, and green electronics (Ahmad et al. 2022; Soleimani et al. 2022). Cellulose fiber (CF) valorization in many of these end-uses requires the swelling, activation, dissolution, functionalization, or disintegration of the hierarchical and recalcitrant complex structures CFs via chemical, mechanical, enzymatic modifications or an integrated approach (Lavoine et al. 2012; Wohlert et al. 2022; Zuppolini et al. 2022). The existing modification treatments often rely on harsh conditions or the utilization of toxic synthetic chemicals, thus necessitating the development of more sustainable alternatives.

Deep eutectic solvents (DESs) represent a versatile chemical class that functions as solvents, reactants, and catalysts (Álvarez et al. 2023; Di Carmine et al. 2021; Sirviö et al. 2015). These solvents are characterized as mixtures exhibiting an actual eutectic-point temperature that is lower than that of an ideal liquid mixture (Martins et al. 2019). They are typically prepared by a facile combination of a hydrogen bond acceptor (HBA), such as quaternary ammonium salts, and a hydrogen bond donor (HBD), such as amines and carboxylic acids. Many DESs are attractive candidates for sustainable material processing owing to their fascinating properties, including low toxicity, high biodegradability, low vapor pressure, and high tunability (Afonso et al. 2023). Specifically, DESs exhibit potential for cellulose modification, including cellulose cationization, as well as the promotion of fiber swelling and nanofibrillation, thereby addressing many limitations of traditional solvents (Ma et al. 2023; Sirviö et al. 2019, 2015; Suopajärvi et al. 2017).

Further, the interactions between non-derivatizing (non-reactive) DESs and CFs are primarily driven by hydrogen bonding and van der Waals forces, which facilitate the disruption of CF architecture and molecular packing, resulting in the loosening of CF cell wall structure or CF dissolution (Alsoy Altinkaya 2024; Li et al. 2017; Wawoczny et al. 2023). Notably, studies revealed that several DES systems induce fiber swelling without reducing the molecular weight of cellulose, demonstrating their efficiency in promoting nanocellulose production or cellulose enzymatic accessibility (Bi et al. 2023; Sirviö et al. 2022). Specifically, a DES system comprising triethylmethylammonium chloride (TEMACl) and imidazole (Imi), TEMACl–Imi, was employed for wood and CF modification, effectively enhancing the cellulose-nanofiber properties (Sirviö and Visanko 2017). However, the mechanism and interactions driving the swelling phenomena remain unknown.

Nuclear magnetic resonance (NMR) spectroscopy represents a valuable tool for investigating molecular structures and behaviors (Keeler 2011). NMR facilitates the real time observation of molecular interactions, capturing atomic-level dynamic processes (Callaghan 2011). Leveraging this capability, NMR offers precise insights into the interactions, binding, and behavior of molecules in diverse environments, making it an invaluable tool for studying complex systems. Additionally, NMR spectra reveal information about chemical shifts, which reflect the electronic environment surrounding specific nuclei, and this enhances the understanding of the structural information of molecules, including their functional groups and molecular interactions. In addition, NMR assesses molecular dynamics through relaxation and diffusion experiments (Callaghan 2011). Furthermore, relaxation times (*T*_1_ and *T*_2_) and diffusion coefficient (*D*), which predominantly reflect the rotational and translational motions of molecules, respectively, provide versatile information on molecular mobility and interactions.

In the context of DES–cellulose systems, NMR experiments have been instrumental to the elucidation of the molecular dynamics and interactions within these complex mixtures (Davies et al. 2010; D’Agostino et al. 2011). For instance, NMR diffusion experiments have revealed the mobility of individual components in DES when confined within bacterial cellulose gels, highlighting differences in the diffusion rates of various solutes (Smith et al. 2020). Furthermore, NMR was used to investigate the dissolution behaviors of cellulose in DES systems, providing insights into the role of hydrogen bonding, as well as the molecular interactions that govern solubility and structural integrity (Jiang et al. 2014; Wang et al. 2016).

In this study, which was based on diverse NMR techniques, including proton (^1^H) and carbon-13 (^13^C) NMR spectra, as well as ^1^H relaxation and diffusion experiments, complemented by several other techniques, including solvatomagnetic solvent analyses, we revealed the molecular interactions in a heterogeneous system comprising CFs and two different isomeric, non-reactive DES system: TEMACl with imidazole (Imi), TEMACl–Imi, and TEMACl with pyrazole (Pyra), TEMACl–Pyra. Imi and Pyra are isomeric forms comprising five-membered cyclic aromatic rings bearing two nitrogen atoms. The study is aimed at interlinking the molecular composition of DES with cellulose–DES interactions and offers valuable insights into cellulose processing in DES systems.

## Experimental section

### Materials

TEMACl (98%) and Imi (98%) were obtained from Tokyo Chemical Industry Co., and Pyra (98%) was obtained from Thermo Scientific. Dry sheets of commercial dissolving softwood pulp (96.2% cellulose, 3.5% hemicelluloses, < 0.5% total lignin, and 0.17% acetone-soluble extractives; Domsjö Fabriker AB, Sweden) were utilized as the CF source. The probes employed for the solvatomagnetic measurements included pyridine N-oxide (95%), and tetramethylsilane (TMS; ≥ 99.9%) from Sigma-Aldrich; and trifluoroacetic acid (TFA; ≥ 99%), 4- fluoroanisole (≥ 97%), and 4-fluorophenol (≥ 99%) from Tokyo Chemical Industry Co. Dimethyl sulfoxide-d6 (DMSO; 99.8%) used as a lock substance in the long ^13^C experiments was purchased from Eurisotop.

### Preparation of the deep eutectic solvents

Both DES systems were prepared in a glass beaker by combining TEMACl with Imi or Pyra (TEMACl–Imi or TEMACl–Pyra, respectively) in a 3:7 molar ratio (Sirviö and Visanko 2017). Thereafter, both mixtures were heated in Memmert universal heating oven at 80℃ until homogenous solvents were obtained.

### Treatment of the cellulose fibers with deep eutectic solvents

The dried pulp sheets were disintegrated in deionized water, following standard procedure (ISO 2004). Afterward, the soaked pulp was collected by filtration, washed thoroughly with technical ethanol, and subjected to additional stirring in ethanol for 30 min. Subsequently, the mixture was re-filtered and dried at 60 °C to obtain the final cellulose material. Following the methodology described in (Sirviö et al. 2015), this cellulose material was subsequently treated separately with the DES systems using a 1 wt.% solid content. This content was considered to be high enough to observe well the DES-cellulose interactions in the NMR experiments and low enough to maintain sufficient mixing.

### Solvatomagnetic method

The NMR experiments for the solvatomagnetic method were performed using a 11.7-T Bruker Ascend Evo 500 spectrometer equipped with a 5 mm broadband observe (BBO) cryoprobe. To determine the hydrogen bond basicity (*α*_M_), 47.55 mg of pyridine-N-oxide was dissolved in 2 mL of TEMACl–Imi and TEMACl-Pyra DESs in an oven for 12 h at 40 °C. Next, the one-dimensional ^13^C spectra were measured at 298 K using, a spectral width of 240 ppm, a relaxation delay of 2 s, 8 scans, and a 30° tip angle. Deuterated chloroform (77.16 ppm) with TMS (0 ppm for ^13^C) was utilized as an internal standard. The C2 (*δ*_2_) and C4 (*δ*_4_) chemical shifts of pyridine-N-oxide were recorded, after which *α*_M_ was calculated using Eq. 7 (Schneider et al. 1992; Teles et al. 2017).

1 $$\alpha_{M} = - 0.15 \times d_{24} + 2.32,$$

where *d*_24_ is *δ*_4_ − *δ*_2_.

Further, the hydrogen bond basicity (*β*_M_) was determined by preparing separate solutions of fluorophenol or fluoroanisole in each DES system at a 1 mg mL⁻^1^ concentration. Briefly, fluoroanisole was dissolved at room temperature, whereas fluorophenol required 12 h of heating at 80 °C to dissolve completely. For the fluorine-19 (^19^F) NMR measurements were measured at 298 K using, a 90° pulse was applied, with a spectral width of 60 ppm, a relaxation delay of 10 s, and 16 scans. Furthermore, TFA (76.55 ppm) was employed as the internal standard. Thereafter, the *β*_M_ values were calculated using Eq. 8(Laurence et al. 2021):

2 $$\beta_{{\mathrm{M}}} = \frac{{\left[ { - {\updelta }\left( {{}_{ }^{19} {\mathrm{F}}} \right)_{{{\mathrm{OH}}}} } \right] - \left\{ {1.009{ }\left[ { - {\updelta }\left( {{}^{9}{\mathrm{F}}} \right)_{{{\mathrm{OMe}}}} } \right] - 1.257} \right\}}}{3.041}$$

where *δ*(^19^F)_OMe_ and *δ*(^19^F)_OH_ are the chemical shifts of the fluorine in 4-fluoroanisole and 4-fluorophenol, respectively.

### Viscosity

The rotational viscosities of both DES systems were measured using a Discovery HR-1 hybrid rheometer (Thermal Analysis Instruments). The flow-sweep procedure was implemented using the following conditions: cone diameter, 40 mm; cone-plate angle, 1.9991°; and step time, 35 s.

### Fiber analysis

The fiber dimensions of the original untreated cellulose pulp and the DES-treated pulps were analyzed using a Valmet FS5 image analyzer, following standard (ISO 2004). The analyses were performed in triplicate, after which the results were averaged.

### Nuclear magnetic resonance experiments

The NMR experiments were performed using a 14.1 T Bruker Avance III 600 spectrometer equipped with a 5 mm BBO probe. The ^1^H spectra were measured at room temperature using a 20-ppm spectral width, 66,000 data points, recycle delay 1 s and 4 scans with a 30° tip angle. The ^13^C spectra were measured using (spectral width, 240 ppm; data points, 42,000; recycle delay 3 s; scans, 70,000; tip angle, 30°). The acquired free-induction decays were processed using the Bruker Topspin software.

Further, ^1^H NMR-relaxation experiments were conducted at variable temperatures, ranging from 293 to 363 K, with increments of 10 K. The implemented temperature-stabilization delay was 15 min. Additionally, spin–lattice *T*_1_ relaxation times were determined using the inversion-recovery pulse sequence (Vold et al. 1968), with four scans, 10 s relaxation delay, and 10 ms–5 s recovery delay with 12 incremental steps. The spin–spin *T*_2_ relaxation times were measured using the Carr–Purcell–Meiboom–Gill pulse sequence (Meiboom and Gill 1958), with four scans, 10 s of relaxation delay, 1 ms of echo time, and 2–2000 incrementally varying number of echoes.

Furthermore, the ^1^H NMR-diffusion experiments were conducted using the pulsed-field gradient-stimulated echo pulse sequence (Tanner 1970), using a gradient-pulse length of 5 ms, a diffusion delay of 0.5 s, a relaxation delay of 11 s, and four scans was 4. The gradient strength increased from 0.01 to 0.5 T/m within 32 linear steps.

The temperature dependence of *D* is represented by the Arrhenius Equation:

3 $$\begin{array}{*{20}c} {D \left( T \right) = D_{0} \exp \left( {\frac{{ - E_{D} }}{RT}} \right),} \\ \end{array}$$

where *D*(*T*) is the diffusion coefficient at temperature *T,* ${D}_{0}$ is the pre-exponential factor, ${E}_{\mathrm{D}}$ is the activation energy for diffusion, *R* is the gas constant, and *T* is the absolute temperature. Taking the natural logarithm, the above equation can be transformed into the following:

4 $$\begin{array}{*{20}c} {\ln D = \ln D_{0} - \frac{{E_{D} }}{RT} \cdot } \\ \end{array}$$

Activation energies (*E*_*D*_) and pre-exponential factors (*D*₀) were obtained by fitting the temperature-dependent diffusion coefficients to the Arrhenius expression (Eq. 3). Nonlinear least-squares fits were performed using Origin software (OriginLab, Northampton, MA, USA) and uncertainties were taken from the standard errors of the fit. This approach follows standard practice for analyzing temperature-dependent diffusion processes (Atkins et al. 2018).

## Results and discussions

### Cellulose-fiber treatment using the isomeric deep eutectic solvents

**Fig. 1:**
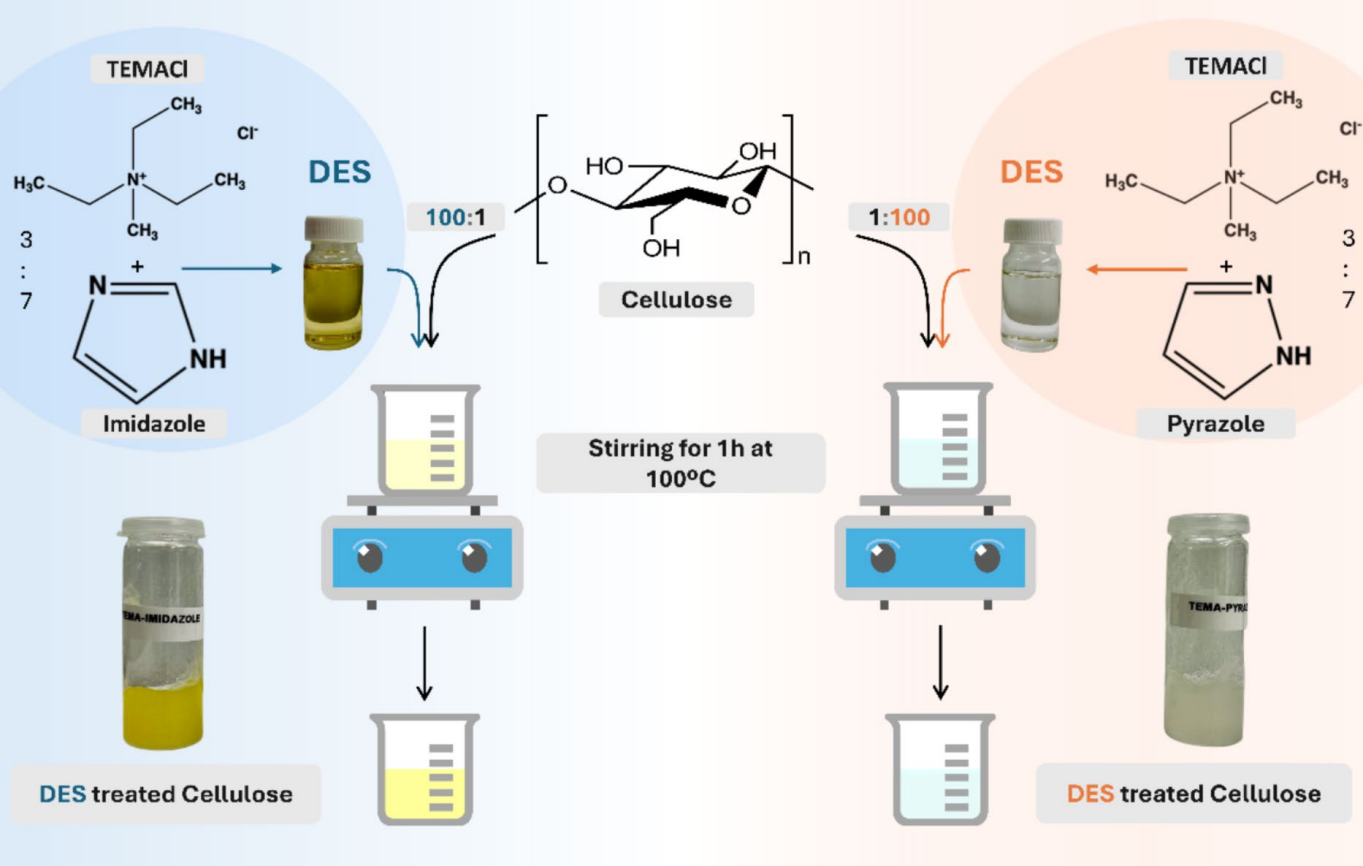
Cellulose fiber treatment with DESs comprising TEMACl–Imi (3:7) and TEMACl–Pyra (3:7)

The as-prepared cellulose-pulp fibers were treated with two different isomeric DES systems: TEMACl–Imi or TEMACl–Pyra at a 3:7 molar ratio to promote CF swelling (Fig. 1). First, pure DES systems were synthesized by heating the components for 1 h at 80 °C, yielding clear, viscous uniform solutions. Both DESs were liquids at room temperature and remained liquid across all temperatures studied. Next, the fibers were immersed in the prepared DESs for 1 h at 100 °C to facilitate the swelling and loosening of its structure without significantly degrading the cellulose-chain integrity (Sirviö et al. 2015).

TEMACl, in the DES complex mixture, coordinates with Imi or Pyra molecules, which are isomers each bearing a five-membered cyclic aromatic ring consisting of two nitrogen atoms, to form a hydrogen-bonded DES complex (Fig. 1). The basicity degrees of Imi and Pyra account for their notable difference, with Imi exhibiting a significantly higher *pK*_*a*_ value (6.95) than protonated Pyra (2.52) (Hu et al. 2010). This difference is attributable to the higher electron density of the nitrogen atoms of Imi, which increases its protonation susceptibility, its resonance stabilization, arising from the resonance between the nitrogen atoms, and its stronger aromatic nature than that of Pyra, owing to the more delocalized electron density in its ring structure. Collectively, these factors enhance the overall stabilization of the energy of Imi (Curutchet et al. 2011). Consequently, Imi tends to form stronger hydrogen bonds owing to distinct nitrogen functionalities, whereas the hydrogen bond formation of Pyra is influenced by its tautomeric equilibrium and electronic effects.

### Solvatomagnetic analysis of deep eutectic solvents

The solvent properties of DES systems, with a focus on hydrogen bonding interactions, were evaluated using the solvatomagnetic method. This technique was chosen for its well-documented high sensitivity in detecting subtle differences between isomeric DES systems. Notably, solvatomagnetic approaches have been shown to provide more accurate and reproducible measurements of solvent hydrogen bonding properties in amphiprotic and ionic media compared to other methods. This advantage is particularly important for complex systems such as DESs, which exhibit strong internal hydrogen bonding and high viscosity (Laurence et al. 2021, 2014).

The solvatomagnetic technique employs NMR-active probes (^13^C and ^19^F nuclei of pyridine-N-oxide and fluorophenol or fluoroanisole in this case) to quantify DES hydrogen bond acidity (${\alpha }_{\mathrm{M}}$) and basicity $({\beta }_{\mathrm{M}})$, particularly in viscous and complex systems where other methods may lack precision.

Our solvatomagnetic measurements (Table 1) yielded slightly higher ${\alpha }_{\mathrm{M}}$ for TEMACl–Imi (4.14) than for TEMACl–Pyra (4.11). Notably, ${\beta }_{\mathrm{M}}$ was also higher for TEMACl–Imi (0.68) than for TEMACl–Pyra (0.65). These values indicate that the Imi-based DES exhibited slightly higher hydrogen-bond-donating and -accepting capabilities than its Pyra-based counterpart. The NH group of Imi is typically more acidic than that of Pyra, and its pyridine-like nitrogen is more basic than that of Pyra, qualitatively supporting findings (Altmann et al. 2015).

**Table 1:** Solvatomagnetic parameters of both DESs: hydrogen bond acidity $({\alpha }_{\mathrm{M}})$ and hydrogen bond basicity (${\beta }_{\mathrm{M}})$

DES	${\alpha }_{\mathrm{M}}$	${\beta }_{\mathrm{M}}$
TEMACl–Imi	4.14 ± 0.01	0.68 ± 0.01
TEMACl–Pyra	4.11 ± 0.01	0.65 ± 0.01

For comparison, the ${\alpha }_{\mathrm{M}}$ values of the TEMACl–Imi and TEMACl–Pyra DESs (4.14 and 4.11) are significantly higher than those reported for the archetypal DES comprising choline chloride and urea (0.85) (Sirviö et al. 2022). This difference may stem from the structural nature of the DES components, as Imi and Pyra are aromatic heterocycles bearing active NH groups that act as strong hydrogen bond donors, thereby potentially contributing to elevated ${\alpha }_{\mathrm{M}}$ values. Conversely, the ${\beta }_{\mathrm{M}}$ values for TEMACl–Imi and TEMACl–Pyra were close to those reported for chloride-based DESs, falling within the range for molecular solvents such, as DMSO, ${\beta }_{\mathrm{M}}$ ≈ 0.76, and methanol, ${\beta }_{\mathrm{M}}$≈ 0.62 (Laurence et al. 2014).

It should be noted that α_M_ and β_M_ are defined from chemical-shift differences and are in principle unbounded. In practice, however, most molecular solvents fall within *α*_M_ < 2 and *β*_M_ < 1, while DESs and ionic liquids can reach considerably higher values (Laurence et al. 2025). The present values near *α*_M_ ≈ 4.1 therefore reflect the unusually strong hydrogen-bond donation capability of the imidazole/pyrazole systems studied here.

### Viscosity analysis of deep eutectic solvents

The rheological properties of a solvent significantly influence its interactions with solids, as high viscosity can inhibit dynamic phenomena, such as solvent penetration of porous fiber materials and surface wettability. Our room temperature viscosity measurements revealed that the TEMACl–Imi DES exhibited higher viscosity (0.16 Pa·s) than the TEMACl–Pyra DES (0.03 Pa·s). Regardless, both DESs exhibited Newtonian fluid behaviors, maintaining constant viscosities across the entire shear-rate range (1–1000 s⁻^1^).

The viscosity values for TEMACl–Imi and TEMACl–Pyra DESs were considerably higher than those of conventional organic solvents, typically ranging from 0.001 to 0.01 Pas. For instance, traditional solvents, such as ethanol and acetone, exhibit viscosities of 0.0012 and 0.003 Pa·s, respectively (Sun et al. 2023). The extant studies revealed that DESs typically exhibit higher viscosities than traditional solvents. For example, reported that most DESs exhibit viscosities greater than 1 Pa·s, with some reaching values that are as high as 4 Pa·s at room temperature. Therefore, the viscosities of TEMACl–Imi and TEMACl–Pyra were determined to be slightly lower than those of traditional DESs.

The observed viscosity differences between the TEMACl–Imi and TEMACl–Pyra DES systems may be due to the hydrogen bonding degrees between their components, which affect the mobility of the solvent molecules. The higher basicity of Imi might enhance DES-complex formation with TEMACl. For example, the higher electron density on the nitrogen atoms of Imi might facilitate hydrogen bonding with the chloride anion of TEMACl.

We did not measure viscosities of DES-cellulose systems, because our rheometer setup is not well suited for suspensions containing solid cellulose fibers. However, the NMR diffusion measurements reported below reflect qualitatively viscosity changes induced by the fibers.

### Alteration of the cellulose fiber dimensions during treatment with the deep eutectic solvents

After subjecting the CFs to DES treatment, we determined their dimensions using an image analyzer. Following the literature (Suopajärvi et al. 2017), an increase in the lateral dimension (width) of the fiber may indicate the alteration of the fiber cell wall structure, as well as DES-induced fiber swelling. The analysis relied on the dimensions of thousands of individual fibers and could reveal insignificant changes in the fiber width (std < 0.1 µm).

Notably, the DES treatment exerted varying effects on the CF width. For instance, TEMACl-Pyra slightly increased the fiber width compared with the original untreated pulp (33.57 ± 0.07 µm vs. 33.09 ± 0.11 µm, respectively). However, TEMACl–Imi treatment notably enlarged the fiber width, 34.17 ± 0.09 µm. Thus, the TEMACl–Imi DES induced significant fiber swelling, potentially owing to the stronger hydrogen bonding capability of Imi.

The increased fiber dimensions, indicating enhanced swelling, do not imply chemical degradation or derivatization. The findings confirmed that the molecular structures of CFs remained stable after the treatment, as demonstrated by their retained crystallinity and insignificant chemical alterations, following DES exposure (Li et al. 2017; Sirviö et al. 2015). Overall, these results indicate that only a slight change in the molecular structure of a DES can significantly impact DES–cellulose interactions and affect CF behavior in the DES.

### Nuclear magnetic resonance analysis of deep eutectic solvent–cellulose fiber interactions

To investigate the molecular interactions between DESs and cellulose, we integrated several NMR techniques. The ^1^H and ^13^C NMR spectra provided information on chemical shifts and signal visibility, allowing us to detect local changes in environments surrounding the DES and cellulose components. Additionally, *T*_1_ and *T*_2_ relaxation measurements were performed to elucidate the effects of the presence of cellulose on the molecular dynamics of DES components, particularly through rotational-mobility alterations. Additionally, NMR diffusion experiments were conducted to explore the random Brownian translational movement of DES components in solutions and determine how this motion impacted cellulose. Together, these experiments offer insights into the strength and nature of DES–cellulose interactions, particularly regarding their hydrogen bonding and swelling behaviors.

**Fig. 2:**
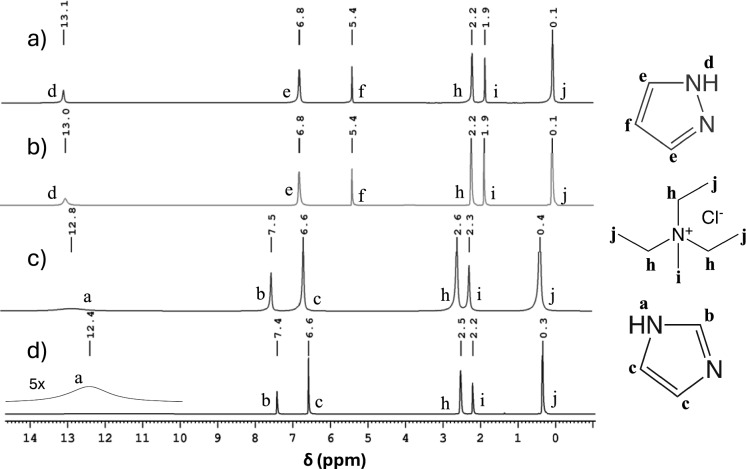
^1^H NMR spectra at 298 K of (**a**) TEMACl–Pyra DES in the presence of CFs, **b** pure TEMACl–Pyra DES, **c** TEMACl–Imi DES in the presence of CFs, and **d** pure TEMACl–Imi DES

The ^1^H NMR spectra of the DES systems are shown in Fig. 2. These spectra only display solvent signals, as cellulose signals were not observed owing to the low cellulose concentration (1% wt) and short *T*_2_ relaxation time due to the slow cellulose mobility. The addition of cellulose to the TEMACl–Imi DES increased the chemical shift associated with the NH proton (peak d) by ≈0.4 ppm (from 12.4 to 12.8 ppm), indicating the hydrogen bonding interactions of cellulose with the NH group of Imi. Conversely, the shift in the corresponding NH peak in the TEMACl–Pyra DES was much smaller, 0.1 ppm, indicating significantly weaker cellulose–NH group interaction, correlating with the reduced cellulose-swelling degree observed in fiber-image analysis.

**Fig. 3:**
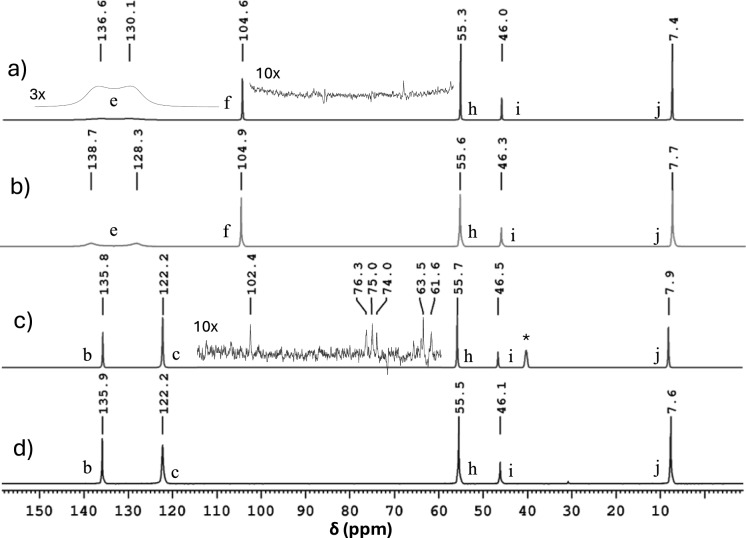
^13^C NMR spectra at 298 K of (**a**) TEMACl–Pyra DES in the presence of CFs, **b** pure TEMACl–Pyra DES, **c** TEMACl–Imi DES in the presence of CFs, and **d** pure TEMACl–Imi DES. The signal of a small amount of DMSO added to the sample for locking during the long (2.5 days per spectrum) ^13^C experiments is represented by * in Fig. 3c (the sample did not include DMSO in the ^1^H experiments)

The ^13^C NMR spectra of the DES–cellulose systems are shown in Fig. 3. Characteristic cellulose-backbone signals are visible at 62–102 ppm (Sasaki et al. 2024) for the TEMACl–Imi DES. However, these signals are absent in the TEMACl–Pyra-DES spectra, despite using the same fiber concentration. The appearance of cellulose signals in the former sample was attributed to TEMACl–Imi-DES induced significant fiber swelling. This swelling enhanced the mobility of the cellulose chains, elongating the ^13^C *T*_2_ relaxation times and narrowing the line widths. Although the ^13^C peaks are not quantitatively comparable due to varying NOE effects and nonquantitative relaxation delays, the peak intensities in Fig. 3c imply that majority of the cellulose chains became mobile due to the DES treatment. Conversely, cellulose signals were not observed in the latter sample, probably because of the shorter *T*_2_ relaxation times and broader line widths associated with the more solid-like, rigid fibers in the TEMACl–Pyra DES system.

**Fig. 4:**
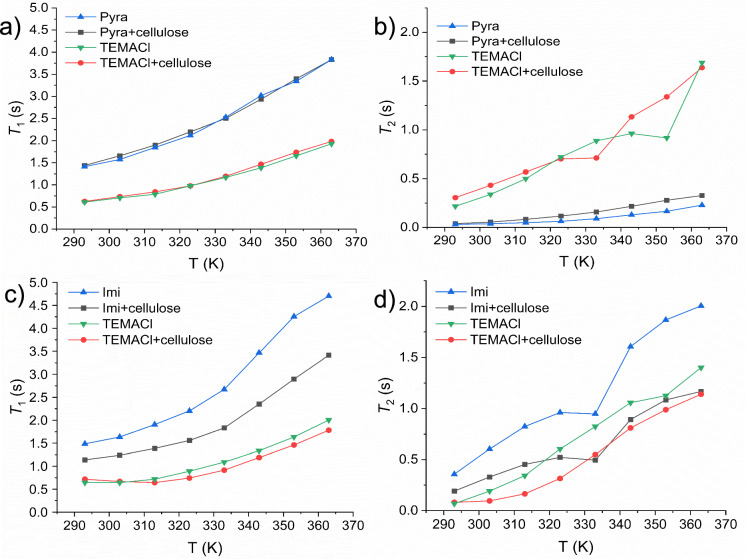
^1^H NMR relaxation times of TEMACl–Pyra and TEMACl–Imi DESs, measured with and without CFs. **a** and **b** show the temperature dependences of *T*_1_ and *T*_2_, respectively, for the TEMACl–Pyra system, and **c** and **d** show the corresponding *T*_1_ and *T*_2_ data for the TEMACl–Imi system. The Pyra and Imi relaxation times were determined using signals **e** and **b** (Fig. 2), respectively. The relaxation times of TEMACl were determined by signal j

The ^1^H NMR *T*_1_ and *T*_2_ relaxation times of the DES–cellulose samples are shown in Fig. 4. The addition of cellulose significantly reduced the *T*_1_ and *T*_2_ relaxation times of the Imi-hydrogen signal b in the TEMACl–Imi DES (Fig. 4c and d). This indicates the significantly reduced (rotational) mobility of Imi, probably because of the hydrogen bonding with cellulose. Contrarily, the relaxation times of the TEMACl-methyl signal j remained largely unaffected by the presence of cellulose, indicating weak or negligible cellulose–TEMACl interactions. The addition of cellulose to TEMACl–Pyra DES did not significantly change the relaxation times of Pyra or TEMACl (Fig. 4a and b), indicating that their interactions with cellulose were considerably weaker than those with Imi. This result correlates with the observed lower swelling capacity of the TEMACl–Pyra DES than that of the TEMACl–Imi system. Imidazole proton b *T*_2_ values show a small local minimum at 334 K (Fig. 4d). Proton b is the non-exchangeable proton attached to the carbon in between the two nitrogens. Hence, the dip cannot arise from the exchange of the proton itself. Similar dip is observed both for the samples with and without cellulose, so it seems not to be cellulose induced. It may be related to the temperature dependent NH proton exchange as well as the formation and breaking of hydrogen bonds, which may slightly modulate the chemical shift of proton b.

**Fig. 5:**
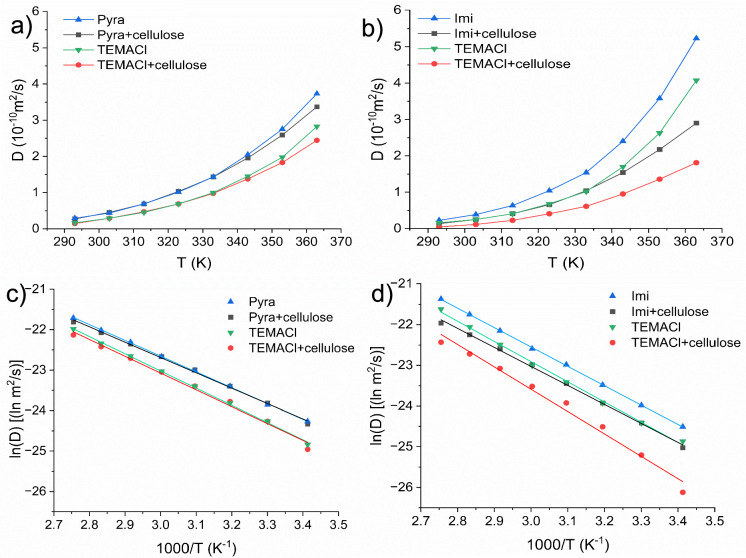
Diffusion coefficients of (**a**) TEMACl–Pyra and **b** TEMACl–Imi with and without CFs. *D* values were determined using the Pyra e, Imi b, and TEMACl j ^1^H NMR signals. **c**, **d** Arrhenius plots of the *D*s and their fittings to Eq. 10 (solid lines)

Figure 5 shows the *D* values of the DES–cellulose systems measured by ^1^H NMR. For the TEMACl–Imi DES, the *D* values of Imi and TEMACl decreased significantly following cellulose addition. This significant decrease in the *D* values reflects the restricted translational motion of both DES components, indicating that the cellulose matrix imposes substantial hindrance, probably via enhanced molecular interactions, such as hydrogen bonding and physical entrapments within the fiber network (Gentile and Olsson 2016). The reduced diffusion can also be attributed to enhanced effective viscosity, as well as the formation of a highly structured environment around the DES components following CF swelling (Li et al. 2017). However, the TEMACl–Pyra DES exhibited almost negligible *D* changes after cellulose incorporation, further confirming much lower fiber swelling and DES–cellulose interactions than observed in the TEMACl–Imi DES. Although bulk viscosity was not directly measured, diffusion coefficients are inherently sensitive to the resistance that molecules experience while moving through the medium. Thus, slower diffusion in the presence of CFs can be interpreted as an effective increase in molecular friction, providing an indirect but quantitative view of viscosity-related changes at the microscopic level.

The ${E}_{\mathrm{D}}$ values (Table 2) obtained from the Arrhenius plots (Fig. 5c and d) represent the energy barrier for diffusion. ${E}_{\mathrm{D}}$ of TEMACl–Imi DES was higher than that of TEMACl–Pyra DES, probably because of the higher viscosity in the former system. Cellulose incorporation did not significantly alter ${E}_{\mathrm{D}}$ of either systems.

**Table 2:** Diffusion activation energies, ${(E}_{\mathrm{D}})$ and pre exponential (*D*_0_) values of DESs and DES–CFs mixtures

TEMACl-Imi			TEMACl-Pyra		
Sample	${E}_{\mathrm{D}}$(kJ/mol)	*D*_0_(m^2^/s)	Sample	${E}_{\mathrm{D}}$(kJ/mol)	*D*_0_(m^2^/s)
Imi + cellulose	38.8 ± 0.7	(1.22 ± 0.13) × 10^–4^	Pyra + cellulose	31.5 ± 0.6	(1.23 ± 0.12) × 10^–5^
TEMACl + cellulose	45.6 ± 2.3	(8.24 ± 0.24) × 10^–4^	TEMACl + cellulose	34.5 ± 1.3	(2.39 ± 0.16) × 10^–5^
Imi	39.5 ± 0.2	(2.57 ± 0.11) × 10^–4^	Pyra	32.4 ± 0.3	(1.89 ± 0.11) × 10^–5^
TEMACl	41.1 ± 0.9	(3.28 ± 0.14) × 10^–4^	TEMACl	35.3 ± 0.5	(3.5 ± 0.12) × 10^–5^

**Fig. 6:**
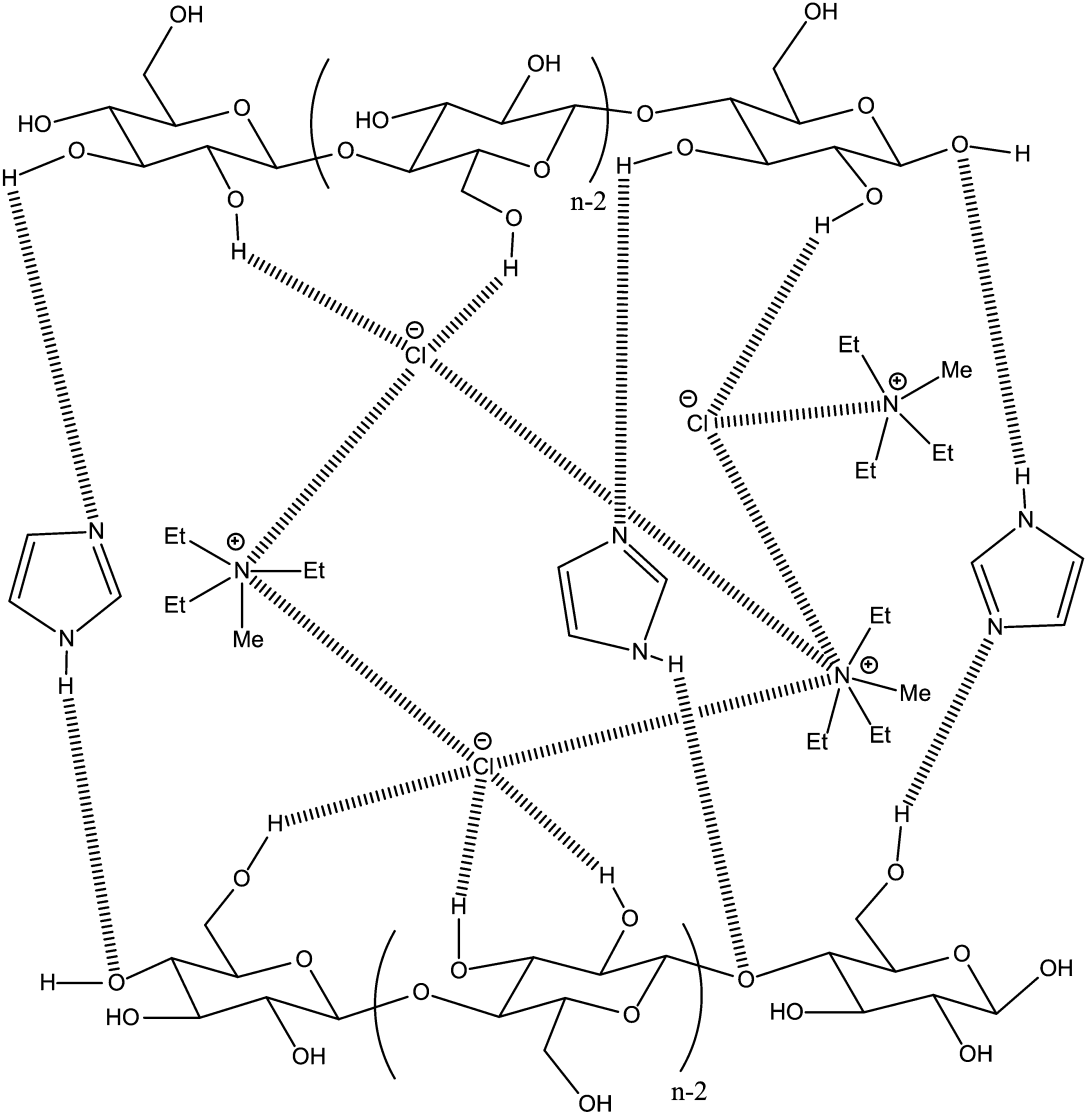
Proposed interaction mechanism between cellulose and the TEMACl–Imi DES system, revealing hydrogen bonding or ionic interaction (dashed lines)

Figure 6 shows a proposed interaction mechanism for cellulose and the TEMACl–Imi DES system based on this study and a findings reported by (Baraka et al. 2024). These interactions accounted for the significant swelling of CFs. Notably, the shift in the Imi NH proton signal (peak a), along with changes in its relaxation time, confirmed the formation of direct hydrogen bonds between Imi and cellulose. Overall, the cellulose interactions with TEMACl–Imi DES disrupted the native hydrogen bonding network within the cellulose matrix, thereby enhancing fiber swelling and increasing accessibility. Conversely, the isomeric TEMACl–Pyra DES displayed a significantly weaker interaction with cellulose, also reflected by reduced fiber swelling. In addition to the role of HBD, the chloride ion in TEMACl was crucial to cellulose swelling through its strong hydrogen-bond-accepting ability and capacity to disrupt the cellulose hydrogen bonding network, as reported by (Baraka et al. 2024).

## Conclusion

This study demonstrated that even slight variations in molecular structures can significantly influence CF–DES interactions, specifically focusing on isomeric DESs comprising TEMACl–Imi or TEMACl–Pyra. The results revealed that the TEMACl–Imi DES exhibited strong interactions with CFs, as evidenced by significant fiber swelling, notable changes in the ^1^H NMR-relaxation times, reduced diffusion coefficients, and distinct cellulose signals in the ^13^C NMR spectra. These interactions were much weaker in TEMACl–Pyra, indicating that Imi facilitates stronger interactions with cellulose than Pyra. This enhanced interaction of Imi-based DESs demonstrates their potential for applications requiring effective cellulose processing and modification. Importantly, our study also shows that diffusion and relaxation measurements by NMR are particularly sensitive for detecting subtle but meaningful differences between chemically similar DESs, demonstrating that molecular-level insight from NMR is essential, as bulk properties alone may not reliably predict solvent performance. Overall, these findings offer valuable insights into the design of environmentally friendly solvent systems, with potential applications in the development of sustainable cellulose materials, thereby advancing green processing technologies.

## Supplementary Information

Below is the link to the electronic supplementary material.

Supplementary file1 (DOCX 109 KB)

## Data Availability

No datasets were generated or analysed during the current study.
