# Supplementary material for: Probing molecular interactions of cellulose fibers with isomeric deep eutectic solvents using NMR spectroscopy
Source: Cellulose (Lond). 2026 Jan 22;33(3):1255–68. doi: 10.1007/s10570-025-06913-0 (PMC12891175; doi:10.1007/s10570-025-06913-0)
Supplement: Supplementary file 1 — Supplementary file1 (DOCX 109 KB) [file 10570_2025_6913_MOESM1_ESM.docx]

**Supplementary information**

Probing Molecular Interactions of Cellulose Fibers with Isomeric Deep Eutectic Solvents Using NMR spectroscopy

Mohan Rangaswamy^a,b,†^, Yashu Kharbanda^a,c,†^, Otto Mankinen^a^, Juho Antti Sirviö^b^, Sarah E. Mailhiot^a^, Mehmet Zafer Köylü^d^, Mateusz Urbańczyk^a,e^, Henrikki Liimatainen^b^, Ville-Veikko Telkki^a^

^a^NMR Research Unit, University of Oulu, P.O. Box 3000, FIN-90014, Oulu, Finland

^b^Fibre and Particle Engineering Research Unit, University of Oulu, P.O. Box 4300,90014, Oulu, Finland

^c^Materials and structures department, Gustave Eiffel University, France

^d^Department of Physics, Faculty of Sciences, Dicle University, Diyarbakir, Turkey

^e^Institute of Physical Chemistry, Polish Academy of Sciences, Poland

Viscosity of the neat DES samples was measured using a rotational rheometer with cone–plate geometry under controlled temperature. Measurements were conducted over a range of shear rates to confirm Newtonian behavior.





**Fig. S1**. Viscosities of TEMACl-Imi and TEMACl-Pyra.

Solvatomagnetic shifts were determined using small, well-defined NMR probe molecules with different functional groups. These probes interact selectively with the solvent environment, allowing changes in polarity, hydrogen bonding, and local interactions within the DES systems to be quantified.

**
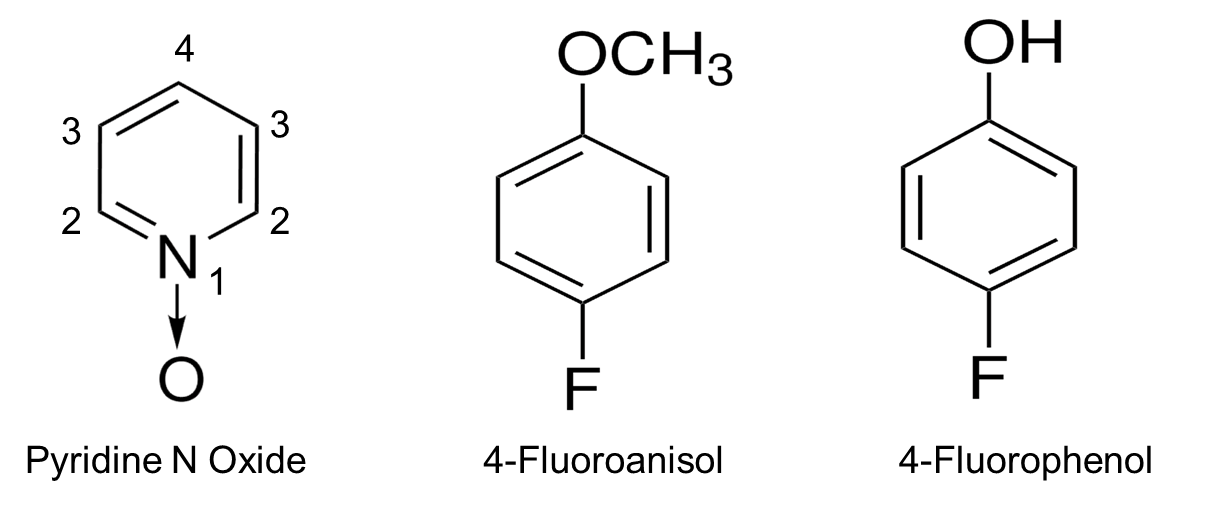
**

**Fig. S2.** Chemical structures of solvent probes used for the solvatomagnetic method.
